# Insertions in the OCL1 locus of *Acinetobacter baumannii* lead to shortened lipooligosaccharides

**DOI:** 10.1016/j.resmic.2014.05.034

**Published:** 2014-07

**Authors:** Johanna J. Kenyon, Kathryn E. Holt, Derek Pickard, Gordon Dougan, Ruth M. Hall

**Affiliations:** a School of Molecular Bioscience, The University of Sydney, New South Wales, Australia; b Department of Biochemistry and Molecular Biology, and Bio21 Molecular Science and Biotechnology Institute, The University of Melbourne, Victoria, Australia; c Wellcome Sanger Trust Institute, Hinxton, Cambridge, United Kingdom

**Keywords:** Lipooligosaccharide, Lipid A, Outer core oligosaccharide, OC locus, ISAba1, ISAba23

## Abstract

Genomes of 82 *Acinetobacter baumannii* global clones 1 (GC1) and 2 (GC2) isolates were sequenced and different forms of the locus predicted to direct synthesis of the outer core (OC) of the lipooligosaccharide were identified. OCL1 was in all GC2 genomes, whereas GC1 isolates carried OCL1, OCL3 or a new locus, OCL5. Three mutants in which an insertion sequence (ISAba1 or ISAba23) interrupted OCL1 were identified. Isolates with OCL1 intact produced only lipooligosaccharide, while the mutants produced lipooligosaccharide of reduced molecular weight. Thus, the assignment of the OC locus as that responsible for the synthesis of the OC is correct.

Lipooligosaccharide (LOS) is a phospholipid–carbohydrate surface structure that is associated with many pathogenic properties of Gram-negative species, including the important nosocomial pathogen, *Acinetobacter baumannii* [1], [2]. It is composed of lipid A, which anchors the LOS in the outer leaflet of the outer membrane, and a core oligosaccharide that extends out from the cell surface. The core consists of several carbohydrates linked together, but is subdivided into inner core and outer core (OC) regions. Genes required for the synthesis of lipid A and the inner core are usually distributed throughout Gram-negative genomes, whereas genes for the OC are usually clustered [3]. In all Gram-negatives studied to date, the OC locus contains multiple genes encoding glycosyltransferase enzymes that catalyse the linkages between the sugars in the OC structure, and may contain genes for sugar synthesis or modification.

We recently reported the identification of two regions in the genomes of *A. baumannii* isolates that include a cluster of genes encoding enzymes for glycosyl transfer and the synthesis or modification of complex sugars [2]. These loci were the only regions found to contain multiple genes associated with surface carbohydrate biosynthesis and to show several different configurations in different genomes. On the basis of all available evidence, the larger locus was unambiguously identified as the gene cluster responsible for the synthesis of the polysaccharide capsule, and was designated the K locus [2], [4], [5]. The smaller gene cluster located between *ilvE* and *aspS* contained multiple glycosyltransferase genes, and must therefore direct the synthesis of the OC component of the LOS. It was designated the OC locus (OCL). Three different OCL forms (OCL1–OCL3) were found in the first ten completed genome sequences [2]. Each contained 9 genes and was between 11 and 12 kb in length.

There was good correlation between the resolved LOS structure of isolate ATCC 19606 [6] and the content of the OCL1 gene cluster that it carries. A second strain, SMAL, produced the same structure [7] and also carries OCL1 (Kenyon and Hall, unpublished). OCL1 contains 5 genes predicted to encode glycosyltransferases that would form linkages in the OC1 structure. OC1 includes glucosamine (D-Glc*p*N) and galactosamine (D-Gal*p*N) sugars, and the *pda1* gene (Fig. 1) encodes an enzyme predicted to deacetylate UDP-*N*-acetyl-glucosamine (UDP-d-Glc*p*NAc) and/or UDP-*N*-acetyl-galactosamine (UDP-d-Gal*p*NAc), producing the UDP-linked form of these products. OCL1 also contains 3 genes without known relatives. One facing in the opposite direction to the remaining genes in OCL1 may encode a glycosyl hydrolase, and the remaining 2 have tentatively been assigned as glycosyltransferases [2].

**Fig. 1:**
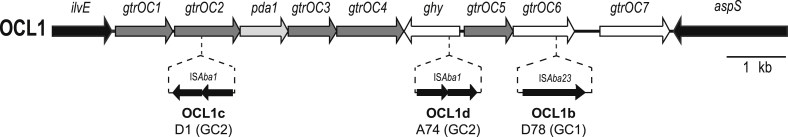
Arrangement of the OCL1 gene cluster and position of IS insertions in Australian *A. baumannii* isolates. OCL1 has been described previously [2]. Arrows represent genes showing the direction of transcription, and gene names are above. Flanking genes are black, genes predicted to encode products required for nucleotide-linked sugar synthesis are light grey, and dark grey genes predict glycosyltransferases. White denotes genes that encode proteins of unknown function with possible functions indicated in the gene names. The *gtrOC6* and *gtrOC7* names were used because a total of 7 glycosyltransferases are needed to construct OC1. IS elements, insertion positions and OC locus names of the resulting OCL1 variants are indicated below.

Here, we have determined the genome sequences of *A. baumannii* isolates belonging to the clinically important global clones, global clone 1 (GC1) and global clone 2 (GC2) from our Australian collection. GC1 and GC2 correspond to CC1 and CC2 in Ref. [8]. Naturally occurring OCL mutants identified among them were used to examine the size of the LOS they produce. This provided experimental evidence confirming the assignment of the OC locus as the region that directs the synthesis of the OC of the LOS.

## 1 Distribution of OCL forms in GC1 and GC2

Whole genome sequences were determined for 82 multiply antibiotic resistant GC1 and GC2 *A. baumannii* isolates from Australian hospitals [9], [10] using Illumina HiSeq. Paired-end reads of 100 bp were assembled using *Velvet*, as described previously [11]. This yielded a median of 116 contigs per genome (median N50, 147 kbp) with an average read depth of at least 70×.

The sequences were examined for the presence of the three reported forms of the OC locus [2]. All 61 GC2 isolates carried OCL1. However, among the 21 GC1 isolates, only 17 carried the OCL1 gene cluster. Three of the remaining 4 GC1 isolates, A85, RBH3 and 6772166 carried OCL3 (GenBank accession KC118540), and one isolate, D13, contained a novel locus designated OCL5 (GenBank accession HM590877) that will be described in detail elsewhere.

## 2 Identification of IS insertions in OCL1

In some genomes (2 GC1 and 11 GC2), OCL1 was not in a single contig, indicating that the locus may be interrupted by a repeated sequence. In each case, this was traced to the presence of an insertion sequence (IS). The assemblies were confirmed by PCR (as described in Ref. [9]) using primers specifically designed to amplify the region that includes the IS, and the products were sequenced for a representative of each group. Three different IS insertions were identified, and the location of these IS elements are shown in Fig. 1. The isolates that carry these interrupted forms of OCL1 are listed in Table 1 together with GenBank accession numbers for one representative.

**Table 1:** Strain information of Australian isolates and position of IS insertions in OCL1.

OC locus^a^	Strains	Global clone	Gene interrupted	IS^b^	Base position^c^	GenBank accession	Reference
OCL1	3208	1	–	–	–	FJ172370	[10]
	A91	2				JN968483	[9]
OCL1b	D78	1	*gtrOC6*	ISAba23 (F)	727	–d	[10]
	D81	1				JN409449	[10]
OCL1c	D1	2	*gtrOC2*	ISAba1 (R)	419	KJ463421	[9]
OCL1d	A74–A82, A84	2	*ghy*	ISAba1 (F)	129	KJ459911	[9]

a OCL1a is interrupted by an ISAba7 insertion sequence (IS) and was designated previously [2].

b F and R indicate that the direction of the transpose gene is the same as, or opposite to, that of *gtrOC1*–*gtrOC6.*

c Bases from the start codon of the interrupted gene.

d The OC locus sequence of D78 and D81 is identical.

Two GC1 isolates, D78 and D81, recovered at Royal North Shore Hospital, Sydney, Australia in the same year included a novel IS that interrupted *gtrOC6* (Fig. 1). This mutant form was designated OCL1b. The IS sequence was deposited in ISFinder (https://www-is.biotoul.fr//is.html) and assigned the name ISAba23. ISAba23 belongs to the IS*5* family, is 1249 bp in length and is bounded by 16 bp inverted repeats. The insertion of ISAba23 has created a duplication of the 5 bp target sequence.

A GC2 isolate, D1, also recovered at Royal North Shore Hospital in 2006, included OCL1c with ISAba1 in the *gtrOC2* glycosyltransferase gene. ISAba1 has previously been shown to increase expression of genes adjacent to its left end (when *insA*/*insB* genes are shown transcribed to the right). This is due to the presence of a strong outward-facing promoter (see Fig S1 in Ref. [12] for references and promoter location). The ISAba1 is oriented such that it directs transcription in the same direction as the majority of genes in the OC locus (Fig. 1). Consequently, it may alleviate polar effects on the transcription of the genes downstream of the IS in *gtrOC2*.

Ten GC2 isolates from Prince of Wales Hospital, Sydney, Australia that were all isolated in 2002 (A74–A82, A84), carried OCL1 with ISAba1 interrupting the *ghy* gene. This gene cluster was designated OCL1d (Fig. 1). ISAba1 is oriented such that the promoter it provides directly opposes the transcription of the preceding 5 genes in OCL1. It is therefore possible that the expression of these genes is prevented or substantially reduced.

## 3 Wild type strains produce only LOS and capsule

Of the 82 Australian isolates examined, five GC2 strains (A91, A93, A94, A96, A97) were found to be closely related to the GC2 isolates (A74-A82, A84) that carry OCL1d, which were recovered three years earlier from the same hospital. Analysis of the sequences revealed these five isolates each carried an intact copy of OCL1, and differed from the OCL1d isolates by 29–51 single nucleotide polymorphisms (SNPs). Hence, these strains provided an essentially isogenic group for the direct comparison of LOS produced by strains carrying OCL1d. The A91 isolate was used as a representative of this group for further analysis.

The surface polysaccharides produced by wild type strains in our collection were examined first. Controls included two strains, SMAL and MG1 (kindly provided by Dr Cristina De Castro, Napoli, Italy), which had previously been shown to produce only LOS [13]. PCR screening demonstrated that both strains carry OCL1, and that MG1 belongs to GC1 (data not shown). In addition, the GC1 isolate, 3208 (recovered in 1997 from the same hospital as D1 and D78) and also carrying OCL1, was also examined. Surface carbohydrates were purified, separated by SDS-PAGE (5% stacking and 16% separating gel) and stained with silver nitrate as described previously [14].

**Fig. 2:**
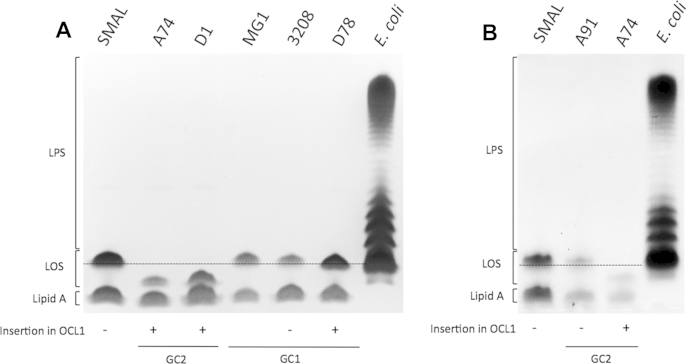
LOS of *A. baumannii* strains carrying OCL1 and OCL1 mutants. LOS and LPS purified extracts were visualized by SDS-PAGE with silver staining. Strain names are indicated at the top and the global clone is shown below. The presence of an IS insertion in the OCL1 locus is also indicated below. The position representative of separated Lipid A, LOS and LPS is shown on the left. The dotted line is aligned to the bottom of the LOS band of SMAL to indicate the position of a complete OC1 on the gel. A. LOS of isolates belonging to GC1 and GC2 that carrying OCL1 or OCL1 with an insertion (Fig. 1). B. LOS of isogenic strains from the Prince of Wales Hospital, Australia compared to the LOS of the control strain, SMAL.

Following SDS-PAGE separation, only two bands were visible in the *A. baumannii* extracts, whereas a lipopolysaccharide (LPS) ladder is observed in the *Escherichia coli* control sample (Fig. 2A and B). Larger bands were not seen in the *A. baumannii* samples when the gel was overloaded. Though some studies have reported that LPS extracted from *Acinetobacter* species cannot be stained using silver nitrate [13], [15], [16], this conclusion is inconsistent with the silver staining of lipid A and LOS observed here. A Western blot was performed on a duplicate of the gel shown in Fig. 2A and immunostained as described previously [13], [15] with the anti-lipid A antibody (MAb A6) used in those studies. Though this antibody should detect lipid A, LOS and LPS, only two bands were detected in the Western blot after mild acid hydrolysis (data not shown), again indicating that no LPS is produced. The smaller band is lipid A and the larger band represents the LOS (lipid A with core oligosaccharide). The failure to detect LPS in any of the *A. baumannii* samples using silver nitrate staining or the MAb A6 anti-lipid A antibody, is consistent with the absence of a *waaL* gene in *A. baumannii* genomes [2], and a *waaL* gene was not found in any of the 82 genomes examined here. Staining with Alcian blue [17] revealed capsule in all *A. baumannii* samples but not *E. coli* (data not shown).

## 4 Strains with IS insertions in OCL1 produce truncated LOS structures

The SMAL and MG1 controls and the A91 and 3208 wild type strains produced LOS bands of the same size (indicated by the dotted line in Fig. 2A). The size of the LOS of the A74 mutant, which represents the ten GC2 isolates that carry OCL1d, is clearly less than that of the controls (Fig. 2A), including the A91 isogenic wild type strain (Fig. 2B). Hence, the ISAba1 insertion in *ghy* in OCL1d (Fig. 1) directly affects LOS synthesis. The LOS of D1 is also deeply truncated, and the band is only slightly larger than that of A74 (Fig. 2A). The size of the LOS of the GC1 mutant, D78, is also reduced, though only slightly, suggesting that the insertion of ISAba23 in *gtrOC6* affects only one of the final linkages in OC1.

## 5 Conclusion

The fact that insertions in three different genes in OCL1 affected the size of the LOS confirms the role of this locus in directing the synthesis of LOS. Determination of the order in which the glycosyltransferases act will require further experimentation.

## Conflict of interest

None.
